# Innovations, Challenges and Future Directions in Nature of Science Research: Reflections from Early Career Academics

**DOI:** 10.1007/s11165-023-10102-z

**Published:** 2023-02-15

**Authors:** Wonyong Park, Alison Cullinane, Haira Gandolfi, Sahar Alameh, Günkut Mesci

**Affiliations:** 1 University of Southampton, Southampton, UK; 2 University of Edinburgh, Edinburgh, UK; 3 University of Oxford, Oxford, UK; 4 University of Cambridge, Cambridge, UK; 5 University of Kentucky, Lexington, KY USA; 6 Giresun University, Giresun, Turkey

**Keywords:** Nature of science, Early career researchers, Research innovation, History of science, Philosophy of science

## Abstract

There has been sustained research interest in the role of early career researchers in advancing the field and the challenges that they face. However, efforts to document lived experiences of researchers working in a specific research area within science education have been scarce. This paper considers the meaning of innovation in the context of nature of science (NOS) research, drawing from a collective reflection of five early career academics from different backgrounds. After discussing the sources of our motivation to innovate in NOS research, we identify four distinct pathways of innovation. These pathways include (1) delving into specific aspects of NOS in greater depth, (2) exploring the interface of NOS and other established research areas, and (3) using NOS to address pressing social issues, and (4) expanding the methodological repertoire of NOS research. We illustrate these four modes of research innovation using examples from our own work. Barriers to early-career innovation such as the absence of NOS in curricula and initial teacher education, the lack of time to engage with practitioners to develop and implement instructional resources, and the underrepresentation of diverse education systems in NOS research literature are discussed.

## Background

Early career academics (ECAs) play a crucial role in advancing disciplines, by bringing in new perspectives and innovating in their research and teaching. This paper considers the meaning of innovation in the context of nature of science (NOS) research and teaching, drawing from collective reflections of five ECAs. Although NOS is established as a distinct research area within science education, there are few reflections documented on the experiences of science educators who research and teach at universities (Akerson et al., 2014), particularly in the early years of their careers. We explore explores innovations, challenges and future directions in NOS research by bringing together our experiences as ECAs. Specifically, it is a result of collective reflections of five ECAs who recently completed their doctoral projects in NOS and have continued to work in NOS and related areas in permanent (tenure-track) academic posts at universities. This paper is an analytical reconstruction of our communication that occurred between October 2021 and June 2022, including written autobiographical reflections, virtual group calls, emails, written reflections and feedback on each other’s writings.

NOS is a meta-perspective about science that includes views from the historical, philosophical, sociological, psychological and practical aspects of science (Galili, 2019; Hodson, 2014). McComas (2004) referred to NOS as the ‘rules of the game’ (p. 25) which have led to the knowledge production and the evaluation of truth claims in the natural world. This definition states that NOS includes learning about how science functions, viewing scientists at work and reviewing their interactions in a community. Lederman (2007) defined NOS as ‘the epistemology of science, science as a way of knowing, the role of scientists, and the values and beliefs inherent to the development of scientific knowledge’ (p. 833). Today, NOS is an established and maturing subfield of science education, with dedicated research strands in major international conferences, and an academic journal specialising in NOS research (*Science & Education*). Although there is a consensus amongst researchers about the importance of NOS, it is known that there are some fundamental disagreements amongst them in terms of the relationship of NOS and scientific inquiry, the domain-general and domain-specific aspects of NOS and a tenet-based versus a holistic approach to characterising NOS (e.g., Erduran & Dagher, 2014; Hodson, 2014; Irzik & Nola, 2014; Lederman, 2007; McComas et al., 1998). While the debate around NOS is beyond the scope of this paper, we appreciate that each approach to NOS has values, and we believe that meaningful and constructive discussion is possible between ECAs from different traditions.

## Collective Writing: Concept, Method and Process

We were inspired by the idea of ‘collective writing’ that was recently advanced by an international group of educational researchers (Peters et al., 2022). Influenced by Guattari’s (2000) analysis of collective subjectivity, collective writing emphasises intertextuality between the writings of multiple authors and the histories, perspectives and ideologies represented in them. This allows the authors to ‘innovate beyond each individual’s ordinary capacity inhere in and arise from the collective give and take, working towards and working through, the issues arising in such collaborations’ (Peters et al., 2022, p. 49). The collective writing approach also fits well with the sustained interest in self-studies (Dinkelman, 2003) and life histories (Goodson & Choi, 2008) in teacher education. This paper is an attempt to capitalise on these methodological approaches to examine the experiences of five ECAs.

This writing project was instigated in October 2021 by Wonyong who, with Alison, invited three NOS ECAs that they knew from various occasions (e.g., conferences, early career researcher events and Twitter), but had no histories of working together for research. The five ECAs represented a range of perspectives on and approaches to NOS and different cultural and geographical backgrounds. Once the group was formed, each member wrote and circulated a short autobiographical essay of two to three pages around questions relating to their entry into NOS research, the innovations sought in their past (particularly doctoral) and current research programmes, the meaning of NOS in their life as an academic, and their views on NOS as a research field. From these essays, Wonyong and Alison used thematic analysis (Braun & Clarke, 2006) to create an initial set of themes that were worthy of further discussion and articulation (e.g. inclusion of NOS in the curriculum, research-teaching relationship) in light of our research question: What are the motivations for, modes of, and barriers to innovation found in our NOS research? A two-hour online group call took place in February 2022 to discuss these themes arising from the essays, as well as other issues around innovation in the context of NOS research. The meeting was moderated by Wonyong and the agenda items were set up by Alison, but all five authors contributed equally to the discussion.

Given that authors and research participants were not separated in our enquiry, the generation and analysis of data occurred in an iterative process. Initial themes generated from essays were used to guide the online call, where new themes arose and were elaborated (Braun & Clarke, 2006). During the two-hour call, connections between our experiences were constantly made and revised by comparing, contrasting, classifying and generalising these experiences, blurring the boundary between data generation and data analysis. We then constructed four sections of the paper on the final, refined themes (Section 4 to 7; Section 3 is for our backgrounds), and two or more authors voluntered to co-write each section. Each group identified subthemes mainly based on the authobiographical essays and the call transcript, using inductive codes. The themes were written up as conversations and then reviewed by all authors. We used the comment function in Google Docs to exchange ideas about the draft manuscript, by suggesting examples that support or challenge the interpretation, references to theorise our experiences, further details to elucidate one’s experience and additional reflections on the issues raised. This process made the feedback not only an analytical step but also a generative one that constantly produced new data. These interactions were then fed back into the manuscript.

In order to represent our voices in a most effective way, we decided to experiment with a new way of collective writing by organising it in the form of conversations. The conversations in this paper are not verbatim transcripts but a way of writing up our collective, empirical findings emerging from the analytical steps outlined above. This presentation, in our view, has several benefits. First, in the same way that qualitative researchers are encouraged to provide verbatim quotes of participants’ words for rich illustration of their experiences (Yin, 2011), the use of conversations can reveal our voices and experiences more powerfully and vividly. Given that the five of us are both the subjects and objects of writing, the format was deemed effective for revealing our lived experiences. Second, we hoped that the conversation format would preserve the plurality of views, approaches and interpretations amongst the five researchers. Although we all research NOS, we trained as doctoral researchers in different research traditions (Wonyong and Alison in ‘family resemblance’, Sahar and Günkut in ‘consensus view’ and Haira in ‘decolonial science and technology studies’) and in different education systems (Ireland, England and USA), and we come from five different countries (South Korea, Ireland, Brazil, Lebanon and Turkey) and currently work in four different education systems (England, Scotland, USA and Turkey). As ECAs, we firmly believe in the value of the coexistence of multiple research paradigms, traditions, perspectives, theories and methodologies (Table 1), and our view is that frequent exchanges of ideas are critical to the development of a research field (Chang, 2012; Giere, 2010). In this sense, the paper can shed light on both the uniqueness of each of us as an ECA and how intellectual exchanges can occur within a heterogeneous group of researchers through conversations. In short, the paper can be seen as a self-study of five ECAs based on their life histories, collectively written in the form of conversations. The conversation starts by sharing our backgrounds as science education researchers and how we entered the field of NOS.

**Table 1:** Summary of our backgrounds

	Wonyong	Alison	Sahar	Haira	Günkut
Country of origin	Korea	Ireland	Lebanon	Brazil	Turkey
PhD location	England	Ireland	USA	England	USA
Current location	England	Scotland	USA	England	Turkey
NOS tradition	FRA	FRA	Consensus	Decolonial STS	Consensus
Entry to NOS	Interest in HPS	Major curriculum reform in Ireland	Interest in HPS, project involvement	Freire’s theory of knowledge and pedagogy	Thomas Kuhn’s philosophy of science
PhD project	Classroom assessment of NOS	Developing preservice teachers’ NOS understandings	Nature of scientific explanation	Decolonial STS for secondary students	Teachers’ PCK about NOS
Major post-PhD projects (see Sect. 5)	NOS in natural and human-made disasters; NOS and pseudoscience	Assessment of scientific methods in school science; the nature of knowledge	NOS within the socio-scientific contexts	NOS in social and scientific emergencies and injustices	NOS instruction based on STEM, PCK, argumentation, outdoor learning

## Starting the Conversation: Our NOS Journeys

Wonyong: Colleagues, thank you very much for being part of this conversation. I think it would be good to start our conversation with a brief summary of our work histories up to and since the doctorate. I will begin. During my undergraduate studies which included initial teacher training, I used to ask many ‘why’ questions about physics and science in general, which led me into the study of history and philosophy of science. After a few NOS-related projects during my master's, my PhD project looked into science teachers’ practices in assessing NOS in the classroom. Both in Korea (where I come from) and England (where I studied and currently work), there is an enduring and powerful tradition of exam-centred education. This means that what is taught and assessed in the classroom is inseparable from what students are tested on (Bakker & Wolf, 2001; Kwon et al., 2017). I wanted to understand how NOS, which has a very different character from scientific content knowledge, could be assessed in the classroom by science teachers. One aspect of my findings was that we need to think about teachers’ abilities to teach and assess NOS separately from those about scientific content knowledge, due to the complicated nature of NOS knowledge (Brock & Park, 2022).
Alison: I came to research NOS through the national circumstances at the time in Ireland (circa 2015), where NOS was introduced to the science curriculum. In a timely meeting with Sibel Erduran, who would become my doctoral supervisor and mentor, I was strongly influenced by her framework of NOS, called the family resemblance approach (FRA) (Erduran & Dagher, 2014). FRA explains science as a cognitive-epistemic as well as social-institutional system, and a ‘family’ of disciplines (e.g., physics, astronomy, geology, medicine) that resemble each other in certain aspects. I found FRA an insightful and promising framework for NOS research in Ireland, particularly at the time of curricular change. It invigorated me and I used the framework to develop preservice teachers’ understanding of NOS (Cullinane & Erduran, 2022). I was so taken by her body of NOS research and found the whole area fascinating. I was astonished that after studying a degree and a master’s in science education; I had not discovered these ideas previously. It fuelled my desire to pursue this in my role as a teacher education researcher. In my postdoctoral work at University of Oxford on Project Calibrate [https://projectcalibrate.web.ox.ac.uk/home], ideas from Erduran & Dagher's NOS framework were used to develop teaching and assessments tools, as well as professional development sessions for inservice and preservice teachers. I plan to continue this line of research while in my new role at the University of Edinburgh with undergraduate science students.
Sahar: My research in NOS started during my master’s studies at American University of Beirut. Much like Wonyong’s story, as a physics graduate with a passion in philosophy, I developed a fascination with science and what it is like. I remember a conversation with a scientist who struggled to answer the question: ‘Have you ever actually *seen* an atom’? It was then when I realised that my fascination with science lay in its uncertainty and imaginative power. During my doctoral program, I worked with Fouad Abd-El-Khalick between 2014 and 2020 and had the chance to be part of several NOS-related projects. Having witnessed first-hand difficulties teachers face when teaching science for understanding, my research started to focus on improving students’ explanations of scientific phenomena. To that end, I developed a framework, the nature of scientific explanation (NOSE), for assessing the type, nature and quality of scientific explanations. I am currently a co-investigator on a grant from the National Institutes of Health in the USA, where I examine teachers’ and students’ views of NOS before and after they participate in a series of lessons and activities about viruses, wastewater, and environmental health. It is my hope that this project gauges how an understanding of NOS shapes opinions about controversial socioscientific issues, such as COVID-19.
Haira: I am a chemistry teacher from Brazil, where Paulo Freire’s (1972) educational thoughts have been influential, especially around how we understand ‘knowledge’. His notion of ‘critical reading of reality’ has been a prominent one, focusing on knowledge as not only about ‘facts’ (the ‘content’) but also about the norms, values and interests involved in the development of any kind of knowledge (including scientific). Inspired by Freire, NOS has been an important aspect of both my practice as a science teacher and my research in science education. During my master’s studies in Brazil, for instance, I explored what our history of colonial science could bring to a more nuanced understanding of how science develops (i.e., NOS) and be linked to political and economic projects. I became fascinated with what non-mainstream examples of scientific development such as the exploitation of natural resources by colonial projects could mean to a more comprehensive ‘critical reading’ (Freire, 1972) of NOS, including to those in the Global North who are not as familiar with those examples as us in the Global South. Hence, in my doctoral research with science teachers in London, we developed decolonial NOS teaching experiences based on these more non-mainstream examples and narratives about scientific development.
Günkut: When I started my master’s studies in the USA, a history and philosophy of science (HPS) course changed my misconceptions about science. I realised that the fundamental knowledge that I had learned for years was actually not in line with currently accepted conceptions of science. Many subjects in science can be learnt and even applied, but unless we think about science and build these subjects on a solid foundation, these subjects will have to collapse and disappear over the years. NOS is the foundation of producing knowledge. For this reason, I desired to understand this field in depth and research NOS in all its aspects and especially contribute to the teaching of prospective science teachers. Therefore, my biggest motivation for raising scientifically literate (Sjöström & Eilks, 2018) generations has been to improve both teachers’ understanding and their NOS teaching practices. In my post-doctoral work, I have also used my experiences of NOS on how NOS could be taught alternatively and more effectively. In this context I researched why some conceptions may be more easily altered than others (Mesci & Schwartz, 2017) and I have been trying to combine NOS teaching with different research areas like STEM, pedagogical content knowledge (PCK), argumentation, and outdoor learning.

## Boredom and the ‘Normal Science’ Status of NOS: Motivation for Innovation

Günkut: It is really refreshing and illuminating to learn about your NOS adventures, having researched it for some years now. When you work on a research topic for many years, you can easily get bored of it. I wonder where exactly such boredom comes from. It could be key to understanding what drives our NOS work.
Haira: I agree. Particularly for NOS, some people ask, ‘Is there anything else to be done about NOS? It is about science, it has been around as a formalised profession and practice for a couple of centuries now, we know how it works’. And I think this question is such an interesting one because it reveals a specific stance on NOS, does it not? As if the field of sciences was static, if it was not dynamic in itself; and it is such an interesting way of looking at science. Does that mean that how scientific development happens never changes, and all is going to be done in the same way in this area? I find it fascinating that some people would think like that, and I quite like how some recent works, such as those around NOS and environmental action (Herman, 2018) and NOS and media literacy (Höttecke & Allchin, 2020), have been pushing against that ‘static’ perspective around science and scientific development. Works like these in the field of NOS research can help us understand that there are different ways of doing this kind of research, including different foci and interests. And that even priorities in research funding will affect the number and diversity of people working in this area, including how they are expected to work, methodologically and in terms of desirable outcomes.
Alison: For me, I saw the repetition and boredom from reading other work and feeling we had come to a standstill in teaching about NOS ideas. When I read NOS research papers, there seemed to be a set list of things to teach about NOS; We know what effective teaching methods are, and we have NOS teaching resources, and there are instruments at our hands for measuring NOS from certain perspectives. As I continued in my journey, I could see new perspectives emerging at the time, and developing examples for the Irish curriculum were invigorating and enabled me to see how ideas could be incorporated to present novel teaching approaches (Allchin, 2011; Cullinane & Erduran, 2022; Erduran & Dagher, 2014; Matthews, 2012).
Günkut: Exactly, Alison, and a related question I pondered was, ‘How can I develop myself as a unique researcher rather than one of the many people who do NOS research, when there is already so much knowledge accumulated on it?’ The considerable achievements in the field of NOS, quite paradoxically, might have made NOS research a bit tedious, repetitive and routine for researchers (Finkielsztein, 2021). It is about the interaction between the status of the field and how we position and develop ourselves as ECAs.
Wonyong: This conversation about boredom, tediousness and researcher identity reminds me of Kuhn’s account of ‘normal science’ (Kuhn, 1962). When we look at NOS scholarship as a whole, despite the coexistence of multiple approaches that Alison mentioned, a significant portion of research focuses on using existing theories to ‘solve puzzles’—for example, using the VNOS questionnaire to assess students’ NOS views across demographic groups and education systems, rather than trying to produce radically innovative theories or discoveries. However, this is not to say that these puzzle-solving activities based on a ‘paradigm’ are unhelpful. Kuhn (1962) observed that the paradigm is beneficial in that working in a well-established tradition can be an essential step to scientific advancement by ultimately allowing researchers to identify anomalies and limitations of the current paradigm and coming up with an alternative.
Sahar: There is evidence to support the maturity of NOS as a field. As of May 2022, the most cited NOS paper written by Norm Lederman’s team (Lederman et al., 2002) has been cited by nearly 3,000 studies according to Google Scholar. The earliest works on FRA (Erduran & Dagher, 2014; Irzik & Nola, 2011) have each also accumulated around 500 citations. Given these numbers, I am not surprised that other science education researchers and we wound up wondering if there was any more to be done, on top of the thousands of studies that are out there. I think such a normal scientific status of NOS research can be key to understanding the boredom as well as seeking potential ways to innovate.

## Four Modes of Innovation in NOS Research

Alison: It seems now that we agree it could be from this ‘normal’ status of NOS research that our innovative approaches to this field, as ECAs, emerged. For example, one important innovation in some of our work seems to involve a ‘convergent’ (Kuhn, 1962) approach to NOS where we look deeply into the individual aspects of NOS within the existing traditions (Table 2). My postdoctoral project, for instance, focused on the nature of scientific methods and using ideas from FRA to develop teaching, assessment and professional development tools. My PhD work was innovative as I brought together theoretical frameworks for the first time and created teaching and assessment tools themed around the categories of the FRA. I also recently published an innovative work looking at the nature of geography (Puttick & Cullinane, 2021), where we used the FRA framework to position these ideas for geography education.

**Table 2:** Four modes of innovation in NOS research found across our work as ECAs

Mode of innovation	Convergent	Divergent		Methodological
Description	Delving into specific elements of NOS	Seeking intersections of NOS and other research areas	Relating NOS to socially and globally acute issues	Employing innovative and non-traditional methods
Examples	Nature of scientific methods, nature of knowledge (Alison) Nature of scientific explanation (Sahar)	NOS and classroom assessment (Wonyong) NOS, teacher professional knowledge & meta-strategic knowledge (Alison & Günkut) NOS and decolonial STS (Haira)	NOS in COVID-19 wastewater project (Sahar) NOS and natural and human-caused disasters (Wonyong)	Collaborative action research, epistemic network analysis (Haira) Micro-level conversation analysis of NOS lessons (Wonyong & Haira)
Sahar: That sounds similar to how I innovated in my study on NOSE. For my dissertation, I developed the NOSE framework that aims to meaningfully assess and construct scientific explanations, which is a subset of NOS. The NOSE framework is among the first attempts to develop a functional framework of scientific explanation guided by underlying philosophical models that is useful for school science teaching and learning (Alameh & Abd-El-Khalick, 2018). It helps researchers in science education assess scientific explanations using a framework specific to explanations, rather than using frameworks that are not designed to assess ‘explanations’ per se. Another goal for the framework is to support teachers, and in turn students, as they construct and evaluate scientific explanations. Using this framework, I have looked at university freshman students, high school science teachers, and scientists’ scientific explanations of everyday phenomena and assessed them using the NOSE framework. I am currently working on scaffolding elementary preservice teachers for meaningfully constructing and assessing age-appropriate scientific explanations.
Günkut: I think that such in-depth examinations of existing NOS-related elements and issues as Alison’s and Sahar’s examples are vital to identifying the limits of our current understanding and exploring new agendas for research, given our position that scientific development is not a simple, linear process outlined in (the) ‘scientific method’. Such a simplistic model of scientific development has been challenged by a number of researchers (e.g. Allchin et al., 2014; Erduran & Dagher, 2014; Harding, 2008; Lederman, 2007).
Wonyong: From our stories, I can see another, perhaps opposite, momentum for innovation, which we might call a ‘divergent’ approach that pushes the boundaries of traditional NOS research. This kind of innovation seems to emerge at the interfaces and intersections of NOS and other established research areas in (science) education. My doctoral project has this aspect, as I attempted to bring together NOS and classroom assessment theory, an interface of which had been seldom explored previously (Hanuscin et al., 2020).
Haira: That is interesting, Wonyong. Did the focus on classroom assessment change the way you view NOS and/or classroom assessment?
Wonyong: That is a good point. In my work, I looked at how high school teachers plan and practise classroom assessment of NOS, both for formative and summative purposes. One of the conclusions was that much of what we know about assessment of *science* may not be sufficient for explaining the assessment of *NOS*, since NOS knowledge and scientific content knowledge are very different. Assessing Newton's second law and assessing how scientific knowledge evolves through a social process are very different activities. Some of Günkut’s work also made this point clear by specifying the elements of NOS PCK that cannot be fully captured by PCK for science (Mesci, 2020; Mesci et al., 2020), as argued by Hanuscin et al., (2011) as well.
Alison: My doctoral work could be an example of seeking the intersections, too. The workshops, the assessments and data collection tools were all based theoretically around FRA, teacher professional knowledge bases (Gess-Newsome, 2015) and meta-strategic knowledge (Zohar, 2007). This approach was innovative because these specific frameworks had not been used before to develop preservice teacher understanding of NOS, although we have seen increasing interest in this area more recently (e.g., Edgerly et al., 2022—albeit not involving FRA as a way of conceptualising NOS). And, at the time FRA was in its infancy and so developing teaching tools with this particular framework was innovative in itself. Haira, would your work about decolonial NOS also relate to this mode of innovation?
Haira: Certainly. My doctoral and current work on bringing decolonial lenses to NOS could be another example of this kind of divergent approach emerging at the intersection of NOS and other research areas. I work on broadening the conceptualisation of NOS for science education by engaging with a more recent scholarship from the field of science and technology studies (STS) around decolonial and feminist theories (Elshakry, 2010; Harding, 2008; Patiniotis, 2013; Sousa Santos, 2018).
Alison: Did your interest in STS also partly stem from decolonisation and the Global South?
Haira: Yes, it is certainly linked to my trajectory of teaching and researching NOS from the perspective of a formerly colonised country in the Global South. My work involves recognising that ‘science was itself built upon a global repertoire of wisdom, information, and living and material specimens collected from various corners of the colonial world’ (Roy, 2018). So, this decolonial STS scholarship means exploring, within NOS, the specific notions of power struggles, oppressions and inequalities in scientific development. For instance, exploitation of peoples and resources within scientific processes (Harding, 2008; Ideland, 2018; Sousa Santos, 2018). Here, scientific developments are understood as contextual, being part of a macro-world that is the same macro-world that different communities across the world experience: science is not merely influenced by decisions made in other spheres such as politics and economy, but it is actually a core part of these spheres, such as seen with the Cold War and the COVID-19 pandemic (Carter, 2017). This approach to NOS does not aim to simply ‘uncover’ these complex socio-historical landscapes, as done by most studies around NOS and, for instance, socio-scientific issues (e.g., Herman, 2018), but make visible the unequal relationships established between different communities across the world within scientific endeavours (Gandolfi, 2021a, b).
Sahar: And I think Haira’s comment about complex links between scientific developments, wider society and different communities across the world is closely connected to another divergent approach to innovation found in our research histories, which is using NOS as a vehicle to understand and address urgent problems, including those related to socioscientific issues, such as the COVID-19 pandemic and environmental emergencies.
Wonyong: Yes, for example, one of my recent interests is ‘disaster education’. In considering disaster risk, vulnerability and resilience in the context of science education, I focus on what disasters as failures of technological systems teach us about NOS (Park, 2020a). Consider the Fukushima nuclear disaster in 2011. It tells us a lot about how human-built technological systems can create new types of risks that we do not fully understand (Beck, 1992), and how science and engineering are utilised to investigate catastrophes and mitigate their impact, and what ethical responsibilities for scientists and engineers are there in preventing and responding to disasters. All these questions are deeply connected to NOS and critical scientific literacy (Sjöström & Eilks, 2018).
Sahar: I agree. Think of the pandemic. Several people said ‘Well, the U.S. Center for Disease Control and Prevention said first no masks and then they changed their minds, so there must be some conspiracy theory’. But, in my head, I am thinking: ‘This is actually how science works’. We are living ‘how science works’ now, and this is why we have (and need) projects around what it is there to learn about NOS emerging from this pandemic? (García-Carmona, 2021; Moura et al., 2021). So we can get some insight into this, as we can explore NOS in such an authentic scenario; We would not be working from ‘far-removed’ contexts, distant from our lives (and that of our students), but from an authentic NOS-related scenario, with all its uncertainties and complexities (Allchin et al., 2014) that help us challenge a naive perception of scientific development as linear, incremental and dissociated from wider societal concerns (Aragón-Méndez et al., 2019; Duschl, 2008; Erduran & Dagher, 2014).
Wonyong: This is where your current project about COVID wastewater came from.
Sahar: Indeed. I am a co-investigator on a grant with academics from the College of Medicine and the college of engineering here at the University of Kentucky around COVID wastewater surveillance project (Chapin, 2022; Nelson, 2022). As part of a large project about testing wastewater for COVID-19, I am leading an effort to develop an environmental health curriculum tied to wastewater surveillance, virology and COVID-19 to be piloted in schools in Kentucky. The focus is to explore students’ and teachers’ views of NOS, using the works of Abd-El-Khalick (2012) and Lederman (2007), in the context of socioscientific issues like COVID-19 within wastewater surveillance.
Haira: Sahar’s project is a great example that reminds me of the relevance that this kind of divergent work around NOS can have in supporting the work of educators in scenarios of socioscientific emergencies even beyond the COVID-19 pandemic, such as challenges facing misinformation and social media (Höttecke & Allchin, 2020; Osborne et al., 2022), environmental emergencies and injustices (Gandolfi, 2022), and around other issues relating to the links between scientific development and social injustices and inequalities, such as (scientific) racism (Ideland, 2018; Sheth, 2019; Willinsky, 2020) and access to the quality health system and medical developments. All these examples really speak to the rich connection between NOS and social justice that still needs further exploration (Hansson & Yacoubian, 2020).
Günkut: And going back to different ways of innovation, I think there is another slightly different type of innovation in our work too, which is not directly about the substance of our research but has more to do with research methodologies. What was particularly evident to me was how most of us switched from those prominent large-scale surveys and interviews (see more in a review of the field by Deng et al., 2011) to small-scale in-depth, participatory case studies and other innovative methodologies.
Haira: That is a good observation. My doctoral research was based on curriculum development with a science teacher under a ‘collaborative action research and inquiry’ approach throughout a whole school year. I aimed not only to support this teacher’s implementation of NOS into his practice, but also to foster his own professional learning and growth by positioning him as an active ‘curriculum maker’ and ‘innovator’ within a divergent intercultural and decolonial approach to NOS teaching (Gandolfi, 2020).
Alison: Action research with teachers is really vital to fields such as NOS, especially when NOS is not highlighted very much in the curriculum (Brock & Taber, 2019; Reiss, 2018). There have been very few studies in the field of NOS adopting that methodology (e.g., Akerson et al., 2010), including the point you make about teachers as curriculum makers within NOS (Gandolfi, 2021a), as also argued by Clough (2018).
Haira: Indeed, Alison. Besides collaborative action research, my data collection and analysis methods around our participant students’ understandings of NOS throughout this experience were also diverse, ranging from the usual questionnaires and interviews to diary writing and group mind-mapping about NOS (Gandolfi, 2021b). And even within the more traditional use of questionnaires, I opted to undertake my data analysis based on ‘epistemic network analysis’ (Peters-Burton, 2015), similar to what you did in your work, Alison (Cullinane & Erduran, 2022; Caramaschi et al., 2022; Erduran et al., 2020). This allowed me to go beyond the sole quantification of isolated, standalone NOS elements and ideas which were used by students to answer the questionnaire, as often done in this field (Deng et al., 2011), and to instead map the diverse ways in which students connected different NOS elements and ideas together to make sense of scientific work in their responses. We then created a ‘network’ of linked ideas about NOS to characterise the nuances in students’ understandings about how science works.
Wonyong: And still on this note around your classroom-based work, Haira, if we look at the current body of NOS literature, it is concentrated on either analysis of policies, standards and curricula (macro-level; e.g., McComas & Olson, 2002; Park et al., 2020; Summers et al., 2019) or empirical investigation of individuals’ views of NOS (meso-level; e.g., Lederman et al., 2019). These are seminal, foundational works that have had a great influence on research and practice and that we as ECAs build our own work on, but there are relatively few studies that offer a close look at ‘NOS classrooms’ (micro-level)—the interactions, discourses and teacher talk that contribute to learners’ meaning-making about NOS. This lack of interest in this micro-level NOS research was raised as early as the 1990s (Kelly et al., 1998), but it still persists in my view, with only very few studies focusing on that area (e.g., Voss et al., 2022) when compared to those focusing on macro- and meso-levels. In my doctoral project, I analysed conversations in NOS lessons through a framework called ESRU (elicit, student response, recognise, and use; Ruiz-Primo & Furtak, 2007), which allowed me to capture the dynamic interactions and discourses within the classroom that led to a co-construction of NOS knowledge. And I know you did something similar in your collaboration with the participant teacher in your doctoral project, Haira (Gandolfi, 2020, 2021b). Also, it is not from one of us, but I have recently learnt about the work of a doctoral researcher who used laboratory ethnography to look into individual scientists’ experiences of NOS (Mohan, 2022), which broadens even more the repertoire of methodologies we can use to study NOS. I look forward to seeing what other innovative approaches will enrich NOS research in the next decades.

## Barriers to Early-Career NOS Research and Innovation

Haira: We have talked about innovation in our work, and different ways of pursuing innovation. I think we all agree that NOS is a fruitful research area within science education, particularly with the recent rise of post-truth (McIntyre, 2018) and trust in science being challenged (Reiss, 2022). But in practice, NOS is a difficult topic to study, is it not? The main challenge for me is that, despite the decades-long discussion about NOS, I still do not feel like it is emphasised much in science curricula or lessons.
Saira: In the USA, the *Next Generation Science Standards* (NGSS Lead States, 2013) only has NOS as an appendix with no clear learning expectations presented in the main text (Akerson et al., 2019). This is certainly a retreat from earlier documents such as *Science for All Americans* (AAAS, 1989) or the *Benchmarks for Science Literacy* (AAAS, 1993) where NOS was more explicitly stressed. If the system does not allow much room for discussing NOS with teachers and students, it is hard for us to undertake empirical research on NOS or incorporate our own work into teacher education practice.
Wonyong: The situation in England is similar. In the 1989 National Curriculum, NOS was one of the 17 ‘attainment targets’ for science, but it is ‘gone’ in the current 2013 National Curriculum (Brock & Taber, 2019; Reiss, 2018; Williams, 2019). The decline of NOS in the USA and England is concerning, particularly given the centrality of NOS knowledge in combating misinformation and disinformation (Osborne et al., 2022). When NOS is not part of the curriculum, it is hard to justify including it in teacher training courses, bidding for grants and conducting research about it.
Alison: The situations of these two countries seem quite contrary to recent reforms in some other countries like Ireland, Italy, Korea and Turkey, where NOS has been introduced as a core element of the curriculum (Caramashi et al., 2022; Kaya & Erduran, 2016;  Park et al., 2020). Countries such as Australia are also proactive in introducing NOS by making explicit references to NOS in the national curriculum (ACARA, 2015; McDonald, 2016). If we want teachers to appreciate the importance of learning NOS, it needs to be part of the curriculum and have a strong foundation in teacher education at both preservice and inservice levels. Once the curriculum includes NOS, it will trickle down to teacher training, classroom instruction and assessment.
Haira: I agree that this visible and consistent presence of NOS in school and higher education curricula is an important element in supporting our work around NOS teaching and learning, especially when working with teachers. For example, I have been embedding findings and theoretical elaborations around ‘decolonial NOS’ emerging from my doctoral research (Gandolfi, 2020; Gandolfi, 2021a, b) into my work with preservice and experienced teachers in England, and I found it to be important to link this work on NOS to what science teachers are expected to teach within the national curriculum, even when the presence of NOS there is diminished.
Sahar: Yes, my work with elementary preservice teachers in the USA is actually done as part of a class that I currently teach on science teaching methods, so NOS is embedded in it. I also advise prospective high school science teachers, and I make sure to engage them in NOS-related discussions as well as hands-on activities, but always with a focus on linking to the existing curriculum.
Haira: Exactly. And we also know from previous research (e.g., Lederman, 2007) that it is not only the lack of space for NOS in the curriculum or the lack of teaching resources that operate as a barrier to teachers’ work in this area, but in several cases, a pervasive professional and subject identity that still values a type of science teaching based on ‘definite knowledge’ (i.e., only one way of answering a problem), memorisation of scientific facts, and teacher-centred lessons (Höttecke & Silva, 2011), which is not necessarily conducive to NOS teaching and learning. That is why I believe that a more sustainable and comprehensive work with NOS in school science also needs to encompass rethinking teacher education to support their own learning about NOS, like what you do in the USA, Sahar. In addition, more than ‘inculcating the need for NOS’ among teachers (Clough, 2018), what we also need is to better support their capacity to keep learning and innovating themselves in this area (Fullan, 2007), so they are not solely dependent on top-down curriculum interventions that do not always come, going back to my point earlier about teachers as curriculum makers. In this area, works such as the one by Herman et al. (2019) on the role of informal support networks for teachers’ work with NOS are very important.
Günkut: This discussion about teachers brings us to something that I very much regret, about the lack of time and motivation for academics to engage with practitioners and develop innovative resources for NOS instruction. It is usually the case that science education researchers are responsible for science teacher education, so it is important for me that my work as a researcher and that as a teacher educator are mutually constructive.
Haira: Indeed. I think all of us identify ourselves as teacher educators, right? I love working with teachers, and many of them are actually interested in NOS, as Brock and Taber (2019) recently observed. I would love to collaborate with NOS researchers like you and to put together a more supportive network for those teachers who want to do more in this area; but, unfortunately, this kind of work is less valued in the current research assessment system. The five of us are in relatively secure positions now, but such a culture would discourage early career researchers on precarious contracts even more seriously.
Wonyong: I can see an interesting tension here between our researcher identity and teacher educator identity within the ‘measurement culture’ of academia (MacPhail et al., 2019; Smith, 2017) and the emphasis on certain types of academic work (high-profile journal publications) over others (practitioner collaboration and resource development) when it comes to appointment and subsequent promotions, which are crucial matters for ECAs. This kind of research productivity pressure for teacher educators seems to be a worldwide phenomenon rather than an issue within a particular institution or higher education system (Guberman et al., 2021; Kusahara & Iwata, 2021). What kind of changes will we need to create more space and time to work with teachers and develop teaching resources with them?
Haira: I think researchers will need to make a stronger case to higher education policymakers about the nature of educational research and the value of developing curriculum and instructional resources, publishing practitioner pieces, and working with practitioners, all of which have a direct impact on educational practice. Alison, I remember some of your research team’s NOS resources were published. How did it come about?
Alison: Yes, I was involved in work in Ireland developing a suite of NOS learning resources based on the FRA framework, samples of which we published as part of an edited volume (Erduran et al., 2020). These came out of the implementation of the new curriculum and publishers saw an opportunity to commission our research centre to produce resources for Irish teachers. This process was great for voicing ideas, as we all had to learn about NOS in order to produce the content each month.
Sahar: I agree that resource development and sharing should be more valued and supported. Publishing in prestigious journals is an important way of advancing knowledge as scholars, but it shouldn’t be taken as the *only* way.

## Discussion and Concluding Comments: The Value of Self-reflection and a Plea for Diverse and Networked Scholarship in NOS

**Fig. 1:**
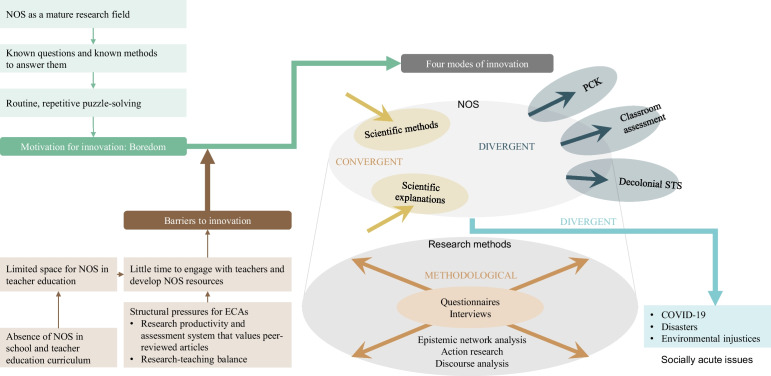
ECAs’ motivations for, modes of and barriers to innovation in NOS research

Unique to our individual stories is the collective meeting of minds as ECAs from across the NOS traditions. This is rarely seen in other publications, and in this article we have explored diverse forms of engagement with NOS through reflecting on our research histories. Grounded on a thematic analysis of those histories and engagements, our enquiry led to identifying three emergent themes that helped us navigate this research area: our motivations for attempting to innovate in NOS research; the modes of innovations we have been engaging with at the content and methodological levels; and the barriers to these kinds of innovations that we have encountered along the way. Figure 1 systematically summarises these themes, associated sub-themes, and how they link to one another. These include unique methodological innovations such as epistemic network analysis and discourse analysis, and novel topics such as understanding the NOS revealed by disasters (e.g., what COVID-19 revealed about international scientific collaboration), to common barrier of space (e.g., in the curriculum) and time (e.g., in teacher education).

As the overall position emerging from this paper, based on our works and hopes as ECAs, we propose the relevance of NOS research to the history and future of science education. As argued by others in this field (Hodson, 2014; Jenkins, 1996), the spirit of and need for NOS has always been there, at least since science was first schooled, with Jenkins (2013) beautifully labelling NOS as the ‘great survivor’ in school science (p. 132). As such, through this article, we hope to continue contributing to the history of NOS within science education by positioning it as an core area of research that can bring new life and ideas to science education. It seeks to respond to the needs of our ever-changing and increasingly complex societies (Hansson & Yacoubian, 2020; Höttecke & Allchin, 2020). We then position the calls we made throughout our conversations alongside Osborne’s presidential address for the U.S. National Association for Research in Science Teaching in 2007, when he pleaded for ‘… bit more time in our armchairs, more time picking over and thinking about what we do—to develop better theories about our goals and values in science education’ (Osborne, 2007, p. 11). A bit more of ‘self-reflection’ around NOS research, as resembled in our reflective discussions, would be helpful in advancing the field.

And within this call for self-reflection, especially around innovative NOS research, we particularly argue for more collaborative, transnational and intercultural approaches to ‘pick[ing] over and think[ing] about’ NOS. Wonyong’s interest in the deep-rooted exam cultures in Korea and England, for instance, shed light on the classroom assessment aspect which had been underexplored in NOS research, while Haira’s Brazilian background has shaped her approach to NOS that is based on Freirean and decolonial ideas about science education.

**Fig. 2:**
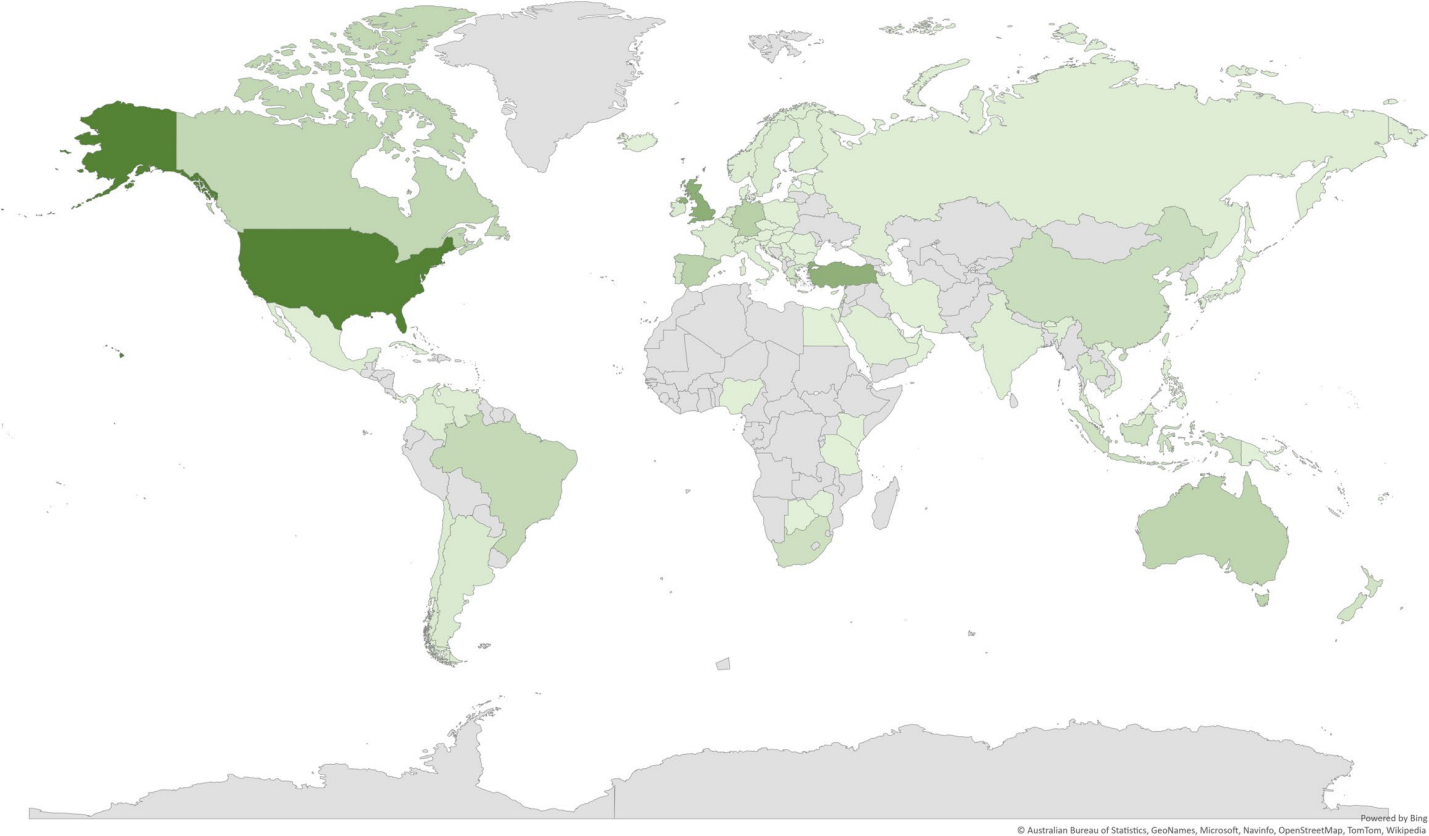
Number of NOS publications by countries (generated from Web of Science data, based on author affiliations)

Nevertheless, currently, NOS research published in the English language is disproportionately focused on a small number of national and cultural contexts. According to the Web of Science data, 36.5% of publications related to NOS research originated from the USA, followed by the UK (7.9%), Turkey (7.7%) and Germany (3.9%) (Fig. 2), with these four countries accounting for 56.0% of current NOS literature, and our current professional affiliations as ECAs are actually a direct representation of that distribution. Given our conversations across this article about the value of heterogeneity and interchange of ideas, we pose that NOS research needs to represent a more diverse array of education systems, each shaped in its own social, cultural, political and historical contexts. As a result, our attempt in this paper is not to say our work is shaking the status quo, ground breaking, generalisable or even representative of wider efforts in this field. Nor did we aim to provide a comprehensive review of current NOS research. As a qualitative, self-reflective study, our findings are subject to the limitations that any case study has about statistical generalisability (Yin, 2013). Despite these limitations, however, we believe collaborations such as the one we engaged with here as ECAs from different traditions can help researchers broaden the way we think about NOS research and science education more generally.

## References

[CR1] Abd-El-Khalick F, Fraser BJ, Tobin K, McRobbie CJ (2012). Nature of science in science education: Toward a coherent framework for synergistic research and development. Second international handbook of science education.

[CR2] Aragón-Méndez MDM, Acevedo-Díaz JA, García-Carmona A (2019). Prospective biology teachers’ understanding of the nature of science through an analysis of the historical case of Semmelweis and childbed fever. Cultural Studies of Science Education.

[CR3] Akerson VL, Cullen TA, Hanson DL (2010). Experienced teachers’ strategies for assessing nature of science conceptions in the elementary classroom. Journal of Science Teacher Education.

[CR4] Akerson VL, Pongsanon K, Weiland IS, Nargund-Joshi V (2014). Developing a professional identity as an elementary teacher of nature of science: A self-study of becoming an elementary teacher. International Journal of Science Education.

[CR5] Akerson VL, Carter I, Pongsanon K, Nargund-Joshi V (2019). Teaching and learning nature of science in elementary classrooms. Science & Education.

[CR6] Alameh S, Abd-El-Khalick F (2018). Towards a philosophically guided schema for studying scientific explanation in science education. Science & Education.

[CR7] Allchin D (2011). Evaluating knowledge of the nature of (whole) science. Science Education.

[CR8] Allchin D, Andersen HM, Nielsen K (2014). Complementary approaches to teaching nature of science: Integrating student inquiry, historical cases, and contemporary cases in classroom practice. Science Education.

[CR9] American Association for the Advancement of Science (1989). Science for all Americans.

[CR10] American Association for the Advancement of Science. (1993). *Benchmarks for science literacy: A Project 2061 report*. Oxford University Press.

[CR11] Australian Curriculum and Reporting Authority (ACARA). (2015). *Australian curriculum: Science F-10*. Commonwealth of Australia.

[CR12] Brock R, Park W (2022). Distinguishing Nature of Science Beliefs, Knowledge and Understandings. Science & Education.

[CR13] Bakker S, Wolf A (2001). Examinations and entry to university: Pressure and change in a mass system. Assessment in Education: Principles, Policy & Practice.

[CR14] Beck U (1992). Risk society: Towards a new modernity.

[CR15] Braun V, Clarke V (2006). Using thematic analysis in psychology. Qualitative Research in Psychology.

[CR16] Brock R, Taber KS (2019). ‘I’m sad that it’s gone’: A case study of teachers’ views on teaching the nature of science at Key Stage 4. School Science Review.

[CR17] Caramaschi M, Cullinane A, Levrini O, Erduran S (2022). Mapping the nature of science in the Italian physics curriculum: from missing links to opportunities for reform. International Journal of Science Education.

[CR18] Carter L (2017). A decolonial moment in science education: Using a socioscientific issue to explore the coloniality of power. Revista Brasileira De Pesquisa Em Educação Em Ciências.

[CR19] Chang H (2012). Is water H2O?: Evidence, realism and pluralism.

[CR20] Chapin, E. (2022). UK's disease detectives use wastewater testing to monitor COVID infection trends. Retrieved 1 May 2022 from https://uknow.uky.edu/research/uks-disease-detectives-use-wastewater-testing-monitor-covid-infection-trends

[CR21] Clough MP (2018). Teaching and learning about the nature of science. SCience & Education.

[CR22] Cullinane, A. & Erduran, S. (2022). Nature of science in preservice science teacher education: Case studies of Irish preservice science teachers. *Journal of Science Teacher Education*. (Online first publication). 10.1080/1046560X.2022.2042978

[CR23] Deng F, Chen D-T, Tsai C-C, Chai CS (2011). Students’ views of the nature of science: A critical review of research. Science Education.

[CR24] Dinkelman T (2003). Self-study in teacher education: A means and ends tool for promoting reflective teaching. Journal of Teacher Education.

[CR25] Duschl R (2008). Science education in three-part harmony: Balancing conceptual, epistemic, and social learning goals. Review of Research in Education.

[CR26] Edgerly, H., Kruse, J., & Wilcox, J. (2022). Investigating elementary teachers' views, implementation, and longitudinal enactment of nature of science instruction. *Science & Education*, 1–25.

[CR27] Elshakry M (2010). When science became Western: Historiographical reflections. Isis.

[CR28] Erduran S, Dagher Z (2014). Reconceptualizing the nature of science for science education: Scientific knowledge, practices and other family categories.

[CR29] Erduran S, Kaya E, Cullinane A, Imren O, Kaya S, McComas WF (2020). Practical learning resources and teacher education strategies for understanding nature of science. Nature of science in science instruction: Rationales and strategies.

[CR30] Finkielsztein M (2021). Boredom and academic work.

[CR31] Freire P (1972). Pedagogy of the oppressed.

[CR32] Fullan, M. (2007). *The new meaning of educational change* (4th ed.). Teachers College Press.

[CR33] Galili I (2019). Towards a refined depiction of nature of science applications to physics education. Science & Education.

[CR34] Gandolfi HE (2020). ‘I didn’t know how that could come to this curriculum’: Teacher’s growth through the development of materials about nature of science. Journal of Science Teacher Education.

[CR35] Gandolfi HE (2021). Decolonising the science curriculum in England: Bringing decolonial science and technology studies to secondary education. The Curriculum Journal.

[CR36] Gandolfi HE (2021). “It's a lot of people in different places working on many ideas”: Possibilities from global history of science to Learning about nature of science. Journal of Research in Science Teaching.

[CR37] Gandolfi HE (2022). Environmental challenges & social justice What can we do about environmental justice through education?. BERA Research Intelligence.

[CR38] García-Carmona A (2021). Learning about the nature of science through the critical and reflective reading of news on the COVID-19 pandemic. Cultural Studies of Science Education.

[CR39] Giere, R. N. (2010). *Scientific perspectivism*. University of Chicago press.

[CR40] Goodson I, Choi PL (2008). Life history and collective memory as methodological strategies: Studying teacher professionalism. Teacher Education Quarterly.

[CR41] Guattari F (2000). The three ecologies.

[CR42] Guberman A, MacPahil A, Ulvik M, Oolbekkink-Marchand H, Vanderlinde R, Smith K, Murray J, Lunenberg M (2021). Teacher educators' professional life stories across four countries: Intertwining personal and contextual effects. Teacher educators and their professional development: Learning from the past, looking to the future.

[CR43] Hansson L, Yacoubian HA, Yacoubian HA, Hansson L (2020). Nature of science for social justice: Why, what and how?. Nature of science for social justice.

[CR44] Hanuscin DL, Lee MH, Akerson VL (2011). Elementary teachers' pedagogical content knowledge for teaching the nature of science. Science Education.

[CR45] Hanuscin D, Khajeloo M, Herman BC, McComas WF (2020). Considering the classroom assessment of nature of science. Nature of science in science education: Rationales and strategies.

[CR46] Harding SG (2008). Sciences from below: Feminisms, postcolonialities, and modernities.

[CR47] Herman BC (2018). Students' environmental NOS views, compassion, intent, and action: Impact of place-based socioscientific issues instruction. Journal of Research in Science Teaching.

[CR48] Herman BC, Olson JK, Clough MP (2019). The role of informal support networks in teaching the nature of science. Research in Science Education.

[CR49] Hodson D, Matthews MR (2014). Nature of science in the science curriculum: Origin, development, implications and shifting emphases. International handbook of research in history, philosophy and science teaching.

[CR50] Höttecke D, Allchin D (2020). Reconceptualizing nature-of-science education in the age of social media. Science Education.

[CR51] Höttecke D, Silva CC (2011). Why implementing history and philosophy in school science education is a challenge: An analysis of obstacles. Science & Education.

[CR52] Ideland M (2018). Science, coloniality, and ‘the Great Rationality Divide’: How practices, places, and persons are culturally attached to one another in science education. Science & Education.

[CR53] Irzik G, Nola R (2011). A family resemblance approach to the nature of science for science education. Science & Education.

[CR54] Irzik G, Nola R, Matthews MR (2014). New directions for nature of science research. International handbook of research in history, philosophy and science teaching.

[CR55] Jenkins EW (1996). The ‘nature of science’ as a curriculum component. Journal of Curriculum Studies.

[CR56] Jenkins EW (2013). The ‘nature of science’ in the school curriculum: The great survivor. Journal of Curriculum Studies.

[CR57] Kaya E, Erduran S (2016). From FRA to RFN, or how the family resemblance approach can be transformed for science curriculum analysis on nature of science. Science & Education.

[CR58] Kelly GJ, Chen C, Crawford T (1998). Methodological considerations for studying science-in-the-making in educational settings. Research in Science Education.

[CR59] Kuhn TS (1962). The structure of scientific revolutions.

[CR60] Kusahara K, Iwata S, Vanderlinde R, Smith K, Murray J, Lunenberg M (2021). Interlude: Teacher educators' professional development in Japan: Context and challenges. Teacher educators and their professional development: Learning from the past, looking to the future.

[CR61] Kwon SK, Lee M, Shin D (2017). Educational assessment in the Republic of Korea: Lights and shadows of high-stake exam-based education system. Assessment in Education: Principles, Policy & Practice.

[CR62] Lederman NG, Abell S, Lederman N (2007). Nature of science: Past, present, and future. Handbook of research on science education.

[CR63] Lederman NG, Abd-El-Khalick F, Bell RL, Schwartz RS (2002). Views of nature of science questionnaire: Toward valid and meaningful assessment of learners' conceptions of nature of science. Journal of Research in Science Teaching.

[CR64] Lederman J, Lederman N, Bartels S, Jimenez J, Akubo M, Aly S, Bao C, Blanquet, … Zhou, Q.  (2019). An international collaborative investigation of beginning seventh grade students’ understandings of scientific inquiry: Establishing a baseline. Journal of Research in Science Teaching.

[CR65] MacPhail A, Ulvik M, Guberman A, Czerniawski G, Oolbekkink-Marchand H, Bain Y (2019). The professional development of higher education-based teacher educators: Needs and realities. Professional Development in Education.

[CR66] Matthews MR, Khine MS (2012). Changing the focus: From nature of science (NOS) to features of science (FOS). Advances in nature of science research: Concepts and methodologies.

[CR67] McComas WF (2004). Keys to teaching the nature of science. The Science Teacher.

[CR68] McComas WF, Olson JK, McComas WF (2002). The nature of science in international science education standards documents. The nature of science in science education.

[CR69] McComas WF, Clough MP, Almazroa H, McComas WF (1998). The role and character of the nature of science in science education. The nature of science in science education.

[CR70] McDonald CV, Abd-El-Khalick F, McDonald CV (2016). Exploring representations of nature of science in Australian junior secondary school science textbooks: A Case Study of Genetics. Representations of nature of science in school science textbooks: A global perspective.

[CR71] McIntyre L (2018). Post-truth.

[CR72] Mesci G (2020). The influence of PCK-based NOS teaching on preservice science teachers’ NOS views. Science & Education.

[CR73] Mesci G, Schwartz RS (2017). Changing preservice science teachers’ views of nature of science: why some conceptions may be more easily altered than others. Research in Science Education.

[CR74] Mesci G, Schwartz RS, Pleasants BAS (2020). Enabling factors of preservice science teachers’ pedagogical content knowledge for nature of science and nature of scientific inquiry. Science & Education.

[CR75] Mohan, A. K. (2022). *Learning in trajectories of participation: Nature of science and temporality in the nature of scientists*. Paper presented at the 2022 NARST International Conference, March 27–30, Vancouver, BC, Canada.

[CR76] Moura CB, Nascimento MM, Lima NW (2021). Epistemic and political confrontations around the public policies to fight COVID-19 pandemic. Science & Education.

[CR77] Nelson, A. (2022). College of education professor takes covid-19 research to Kentucky schools. https://education.uky.edu/college-of-education-professor-takes-covid-19-research-to-kentucky-schools/

[CR78] NGSS Lead States. (2013). *Next generation science standards: For states, by states*. National Academies.

[CR79] Osborne J (2007). In praise of armchair science education. E-NARST News.

[CR80] Osborne J, Pimentel D, Alberts B, Allchin D, Barzilai S, Bergstrom C, Coffey J, Donovan B, Kivinen K, Kozyreva A, Wineburg S (2022). Science education in an age of misinformation.

[CR81] Park W (2020). Beyond the ‘two cultures’ in the teaching of disaster: or how disaster education and science education could benefit each other. Educational Philosophy and Theory.

[CR82] Park W, Wu JY, Erduran S (2020). The nature of STEM disciplines in the science education standards documents from the USA. Korea and Taiwan. Science & Education.

[CR83] Patiniotis M (2013). Between the local and the global: History of science in the European periphery meets postcolonial studies. Centaurus.

[CR84] Peters, M. A., Besley, T., & Arndt, S. (2022). Experimenting with academic subjectivity: Collective writing, peer production and collective intelligence. In M. A. Peters, T. Besley, M. Tesar, L. Jackson, P, Jandrić, S. Arndt & S. Strum (Eds.), *The methodology and philosophy of collective writing* (pp. 38–54). Routledge.

[CR85] Peters-Burton EE (2015). Outcomes of a self-regulated learning curriculum model: Network analysis of middle school students' views of nature of science. Science & Education.

[CR86] Puttick, S., & Cullinane, A. (2021). Towards the nature of geography for geography education: An exploratory account, learning from work on the nature of science. *Journal of Geography in Higher Education*. (Online first publication).

[CR87] Reiss MJ (2018). Beyond 2020: Ten questions for science education. School Science Review.

[CR88] Reiss MJ (2022). Trust, science education and vaccines. Science & Education.

[CR89] Roy, R. D. (2018). Decolonise science – Time to end another imperial era. *The Conversation*. https://theconversation.com/decolonise-science-time-to-end-another-imperial-era-89189

[CR90] Ruiz-Primo MA, Furtak EM (2007). Exploring teachers’ informal formative assessment practices and students’ understanding in the context of scientific inquiry. Journal of Research in Science Teaching.

[CR91] Sheth MJ (2019). Grappling with racism as foundational practice of science teaching. Science Education.

[CR92] Sjöström J, Eilks I, Dori YJ, Mevarech ZR, Baker DR (2018). Reconsidering different visions of scientific literacy and science education based on the concept of Bildung. Cognition, metacognition, and culture in STEM education.

[CR93] Smith J (2017). Target-setting, early-career academic identities and the measurement culture of UK higher education. Higher Education Research & Development.

[CR94] Sousa Santos B (2018). The end of the cognitive empire: The coming of age of epistemologies of the South.

[CR95] Summers, R., Alameh, S., Brunner, J., Maddux, J. M., Wallon, R. C., & Abd-El-Khalick, F. (2019). Representations of nature of science in U.S. science standards: A historical account with contemporary implications. *Journal of Research in Science Teaching, 56*(9), 1234–1268.

[CR96] Voss S, Kruse J, Kent-Schneider I (2022). Comparing student responses to convergent, divergent, and evaluative nature of science questions. Research in Science Education.

[CR97] Williams, J. (2019). T*he nature of science in science education: A case study of the development of the nature of science in the National Curriculum for science 1988 – 2010.* PhD thesis, University of Sussex.

[CR98] Willinsky J (2020). The confounding of race in high school biology textbooks, 2014–2019. Science & Education.

[CR99] Wilson, K., Georgiou, H. & Mills, R. (this issue). Editorial.

[CR100] Yin RK (2011). Qualitative research from start to finish.

[CR101] Yin RK (2013). Validity and generalization in future case study evaluations. Evaluation.

[CR102] Zohar A, Erduran S, Jiménez-Aleixandre MP (2007). Science teacher education and professional development in argumentation. Argumentation in science education.

[CR103] Gess-Newsome J, Berry A, Friedrichsen P, Loughran J (2015). ‘A model of teacher professional knowledge and skill including PCK: Results of the thinking from the PCK summit. Re-examining pedagogical content knowledge in Science Education.

